# Students’ perspectives on racism and anti-racism in physical education: a systematic review

**DOI:** 10.3389/fsoc.2024.1374277

**Published:** 2024-05-22

**Authors:** Lucas Abel, Annette Chidinma Galle, Laszlo Ziehmann, Tobias Vogt

**Affiliations:** Institute of Professional Sport Education and Sport Qualifications, German Sport University, Cologne, Germany

**Keywords:** discrimination, sport, intersectionality, education, school, teaching

## Abstract

Sports, with their various social manifestations, exhibit racist structures and incidents. Physical education (PE) has the potential to serve as an environment to combat racism, but it can also perpetuate and (re)produce racist attitudes and behaviors. This study aimed to conduct a systematic review of national (German) and international literature concerning racism and anti-racism within the context of PE specifically from a students’ perspective. The research methodology followed the Preferred Reporting Items for Systematic Reviews and Meta-Analyses (PRISMA) standard and encompassed a four-step process: (1) searching 11 electronic databases using 70 keyword combinations in both German and English; (2) selecting studies based on five predetermined inclusion criteria; (3) evaluating the quality of selected studies using established appraisal tools; and (4) conducting descriptive and template analyses. Of 5,213 publications, 16 met the inclusion criteria, demonstrating diverse theoretical frameworks and methodological approaches. Five themes were constructed: “How racism is understood” (1); “What students experience,” encompassing discriminatory incidents in PE, sports, and daily life classified as racial stereotypes, prejudices, and everyday racism (2); and “What physical education teachers (3)/Institutions (4)/researchers (5) can and should do.” These themes provided recommendations for teachers, institutions, and researchers, including training and curriculum reforms. While valuable international literature was identified, no German PE specific publications were found emphasizing the necessity of a local (German) survey to comprehend students’ experiences, knowledge, and potential for anti-racism efforts. Such insights are crucial for shaping teacher-related training programs and policy demands in an informed and targeted manner.

## 1 Introduction

Sports in all their social forms are characterized by racist structures and incidents (Hylton, 2017). This ranges from discriminatory behaviors to assaults in professional and amateur sports in the youth sector. Examples are microaggressions, name-calling, insults, and exclusion and racial discrimination on a structural level (Glaser, 2008). During its country-specific monitoring activities, the European Commission against Racism and Intolerance (ECRI) notes that racism in sports has manifested itself in many ways. Moreover, there is a tendency to underestimate racist acts in sports (European Commission against Racism and Intolerance, 2009). These assessments are consistent with those of studies in sports science and practice (Love et al., 2019). Similar to sports, PE frequently portrays an appearance of “colorblindness,” resulting in a minimization or disregard of ethnic and cultural diversity (Barker et al., 2014). Children come into contact with sports not only as observers in stadiums and the media, but also as players in PE and sports clubs (Glaser, 2008). According to the European Commission, sports can be an effective medium for strengthening social cohesion and values such as fair play, mutual respect, and tolerance (European Commission against Racism and Intolerance, 2009). Racism is a challenge for society and working critically on it is inevitable in education (Ledesma and Calderón, 2015).

PE can be an effective way to promote participation in sports and education through sports (Arnold, 1979). In Germany, PE focuses particularly on these two major objectives: “Education toward sport (movement-oriented) and education through sport (personality-oriented).” These objectives are closely interlinked in German PE (Wibowo et al., 2022). However, since Germany has an underlying structural problem with racism, which is also reflected in the school system (Fereidooni and Simon, 2020), it is reasonable to assume that the two major objectives, as well as PE itself, are affected by racism in various forms. This has led to the necessity of working critically toward anti-racism in PE, preventing racism from being consciously or unconsciously (re)produced during lessons. This can be achieved by deconstructing the phenomena and reflecting on them in an anti-racist manner. Students must be empowered to think and act critically against racism. In this study, the term “students” is understood in the school context. Children and young people can learn to deconstruct racism in a culture of movement, play, and sports, developing their ability to act. So far, critical racism content has not been widely disseminated in German PE curricula or university teacher training (Kultusministerkonferenz, 2023).

Students’ perspectives are essential in teaching research because they perceive teaching and learning first-hand. Their perspectives provide valuable information about their achievements and challenges. By including students’ perspectives in research, researchers can gain a more complete and accurate understanding of the teaching and learning processes (Rudduck and Flutter, 2000). Involving students in the research process can promote their engagement and ownership of their education, leading to better learning (Weimer et al., 2014). Furthermore, students’ perspectives can help ensure that the research has relevance and real-life applicability (Bovill et al., 2011).

These challenges have led to the need for anti-racist PE. While previous research has found literature dealing with this topic internationally, there are very few studies conducted in Germany. No systematic review has been conducted yet. While the field is diverse, it is also highly complex and subject to different local manifestations of the global phenomenon of racism, which are controversially debated. Achieving a comprehensive understanding requires the conduct of systematic reviews to enable evidence-based decision-making for future efforts. This study aimed to fill this gap and answer the following question: to what extent and of what quality are national and international publications available in the context of anti-racist PE from the students’ perspective? We also examined whether recommendations for actions that benefit anti-racism PE can be drawn from past publications.

### 1.1 Theoretical framework

Racism is a global phenomenon that manifests itself locally different (Babacan et al., 2020). For reasons of manageability and objectivity, only publications that speak of racism by name were included in the process of this systematic literature research. Nevertheless, this article is based on the fundamental assumptions of the Critical Race Theory (CRT). This concept was developed by Black scholars in the US in the late 20th century to challenge legal systems that perpetuated racial inequalities and white supremacy, emphasizing race as a social construct (Crenshaw et al., 1995). Through the lens of intersectionality in CRT the recognition of different types of oppression experienced by individuals with multiple marginalized social identities, race can be understood as a multifaceted construct (Crenshaw, 1989). CRT integrates an intersectional lens of analysis, acknowledging the interconnected nature of social categorizations such as race, class, gender, sexuality, ability, and other forms of identity and oppression within its framework (Gillborn, 2015). Building on Gillborn’s (2005) perspective, while CRT’s overarching theoretical framework was crafted for the US, it can still be applied to comprehend the mechanisms of racism in a European context. CRT is successfully adapted to find use in education (Taylor et al., 2022) pointing out that power relations within social systems are maintained through the construction of race and racism in society and institutions such as schools (Sleeter, 2016). Also, in PE and Physical Education Teacher Education (PETE) scholars strive to challenge successfully white supremacy, race and racism utilizing CRT (Clark et al., 2019; Blackshear, 2020, 2022; Clark, 2020). Even if this article reports on positive stereotypes in the context of experiences of racism, the understanding is, nonetheless, that there is no such thing as positive racism but rather that racism is always considered negative. Racism research in Germany shaped through the history of the country does not only focus on anti-black racism. For example, the “German Center for Integration and Migration Research” forms six focus groups of racialized people in the “National Discrimination and Racism Monitor”: Black people, Muslims, Asians, Sinti and Roma, Jews and Eastern Europeans (Seidler, 2022). As authors, we incorporate this perspective into the thematic analysis.

## 2 Methods

### 2.1 Author’s positionality and perspective

Before presenting the research methods, it is important to share the perspective of the author. This is not only because template analysis was used as a tool for analyses, but also to enable others to fairly assess the credibility and reliability of our findings. As the first author of this paper, I have written this publication from the perspective of a White male who has never experienced racial oppression, but rather profited from racial privilege. According to Sow (2018), racism is not an issue about the Black community, but rather an insufficient critical reflection of White society. I wish to use my privileges and consider it my duty to critically think and act against racism. To achieve this, I want to become an Ally. Being an Ally means to constantly self-reflect on my privileges and to critically question my position and perspective. Further in line with (Williams, 2019) being an Ally is understood to be actively anti-racist and not just being passively against it. This study was deliberately written to avoid (re)producing conscious and unconscious racism. The second author identifies herself as a Black woman. The third and fourth authors identify themselves as White males. All corresponding authors are German citizens.

### 2.2 Search strategy equations

The search was conducted in nine electronic national (German) and international databases (“Surf,” “FIS,” “BASE,” “Web of Science,” “Scopus,” “The Lens,” “African Journals OnLine,” “DOAJ,” and “ERIC”), using 70 keyword combinations that consisted of two exposures (Table 1). The two exposures were connected by the operator AND, and the keyword combinations by the operator OR.

**Table 1:** Exposures 1 and 2.

Exposure 1	Exposure 2
“Physical education,” “school sport,” “physical education teacher training,” “physical education workshop,” “physical education advanced training”	Racism, “racism research,” “racist experience,” “racism critique” “anti-racism,” “critical whiteness,” “critical race theory”

**Figure 1:**
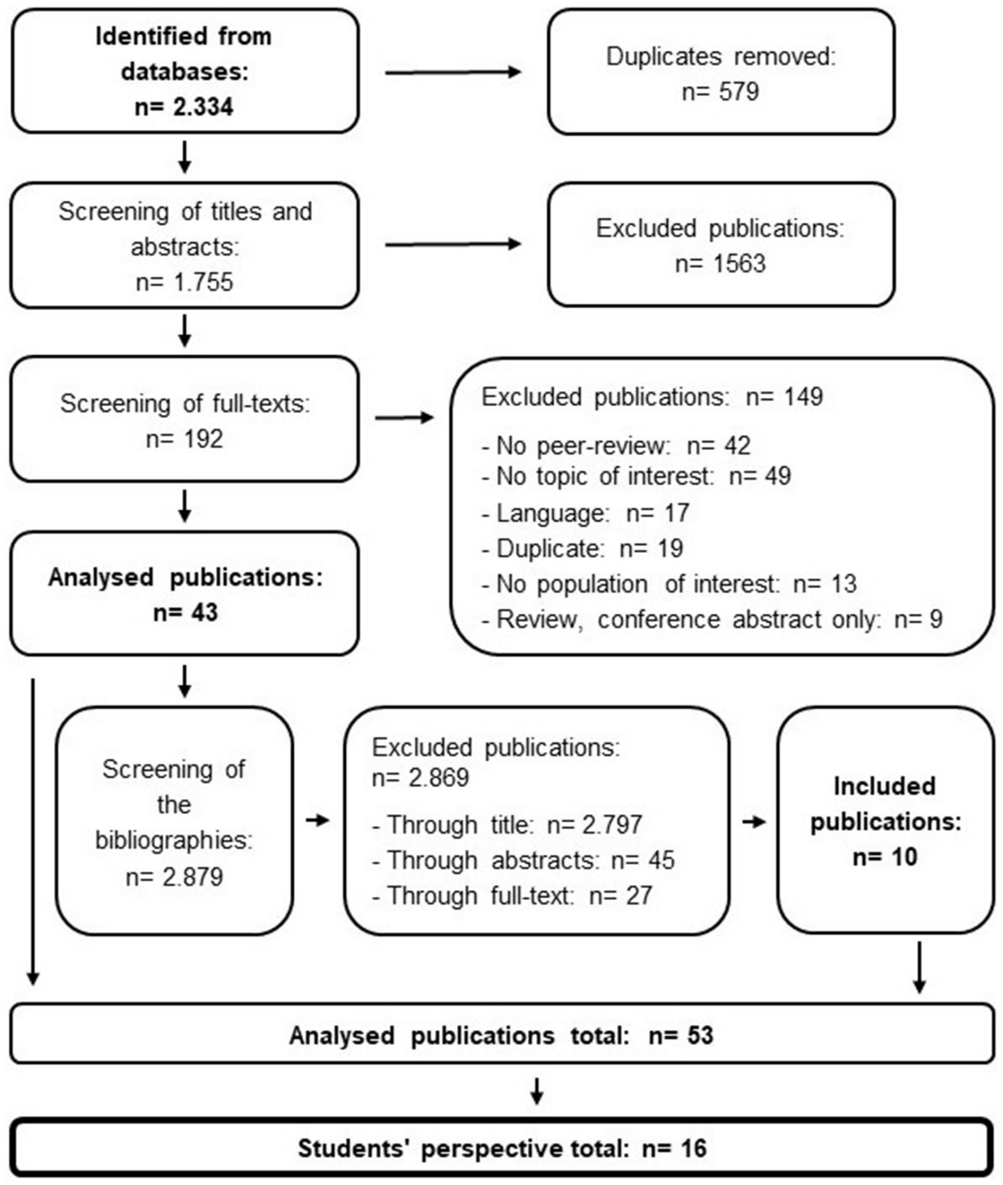
Flowchart of systematic review.

The search terms were translated into German. All databases were searched using 35 English and 35 identical German-translated keyword combinations. There were no limitations regarding the date of publication. Studies potentially relevant to this review were screened according to the following selection criteria in accordance with the Preferred Reporting Items for Systematic Reviews and Meta-Analyses (PRISMA) standard (Page et al., 2021): (a) the publication was published in an international peer-reviewed journal; (b) the publication was published in English and/or German; (c) the study population comprised students, PE teachers or PE teacher training students; (d) the publication discussed racism or anti-racism in PE; and (e) all forms of racism were included as long as they were identified as racism by the publishing author. Duplicates were screened out. Studies were selected by reviewing titles and abstracts identified in the search. Two researchers independently reviewed full-text studies of potential relevance for eligibility. Any differences in article selection were resolved through discussion. A total of 2,334 studies were identified, and 579 duplicates were removed during the literature search. Of the remaining 1,755 publications, 1,563 were rejected because they did not meet the inclusion criteria, leaving 192 potentially relevant studies. The full texts of these studies were reviewed further. Forty-three articles were included in this systematic review while 149 were rejected. The bibliographies of the included articles were checked for other potentially relevant publications. A total of 2,879 titles were reviewed, of which 10 additional full texts met the inclusion criteria. The final selection included 53 studies. After the analysis, three different perspectives on racism and anti-racism in PE were identified. All publications included at least the students’ perspectives, although multiple perspectives were covered, including teachers’ and researchers’ perspectives. We reviewed 16 studies that included students’ perspectives. Figure 1 shows the flowchart of the sampling procedure.

### 2.3 Assessment of study quality

The quality of all selected qualitative publications was identified using the Critical Appraisal Skills Programme (CASP, 018), which is specific to qualitative investigations. A mixed methods appraisal tool (Hong et al., 2018) was used to identify the quality of publications using a quantitative approach. Each item on the applicable checklist used to evaluate each publication had either “yes” or “no” as an answer. Each study received a quality rating (QR) based on this assessment. However, the quality of the listed theoretical publications was not evaluated. For qualitative publications, a study was rated as low quality (LQ) if 0–3 questions were answered by “yes,” medium quality (MQ) if 4–6 questions were answered by “yes,” and high quality (HQ) if 7–10 questions were answered by “yes.” For quantitative publications, a study was rated as LQ if 0–3 questions were answered by “yes,” MQ if 4–5 questions were answered by “yes,” and HQ if 6–7 questions were answered by “yes.” The criteria of the evaluation checklists included the validity and quality of the results. While these two checklists do not provide a rating system themselves, the rating mentioned in this study was adopted from Silva et al. (2021) who also conducted a systematic review. The quality of the studies was independently assessed by two reviewers. A consensus meeting resolved any disagreements between the reviewers.

### 2.4 Data analysis

The following characteristics were summarized for each publication: title, author(s), country, year of publication, objectives, methodology, and quality assessment (Table 2). All 16 articles were analyzed using template thematic analysis (King, 2017). Themes were constructed based on the assumptions of Braun and Clarke (2019) who employed reflexive thematic analysis. As authors, we actively shaped the themes, influenced by our perspectives, attitudes, and the long-term goal of developing a teacher training module. While the topics were more summary-like and less interpretative, the template analysis approach was chosen due to pre-existing theme ideas and the hierarchical assignment of text sections to codes and sub-codes. Constant refinement and expansion of the template occurred during data familiarization, allowing for a conceptual understanding of the dataset.

**Table 2:** Results of the systematic review.

Title	Author(s) (country)	Year	Objective	Methodology	Quality rating
Sport, ethnicity and racism: the experience of Asian heritage boys	Brendon McGuire and David Collins (UK)	1998	“Against the backdrop of these various concerns, this paper contributes to knowledge of issues surrounding the participation of Asian heritage boys in physical education and sport. Through the use of interviews and observation, the research focuses on the experiences of primary and secondary age boys within four inner-urban schools in the North West of England” (McGuire and Collins, 1998).	Qualitative	MQ
A reconceptualization of physical education: the intersection of gender/race/social class	Laura Azzarito and Melinda A. Solomon (US)	2005	“Because schooling should carry the responsibility of educating children to adopt and maintain a physically active lifestyle, the most prominent physical education curriculum in the United States, the sport-based physical education curriculum, requires the reconceptualization of current practice” (Azzarito and Solomon, 2005).	Theoretical work	
A poststructural analysis of high school students’ gender and racialized bodily meanings	Laura Azzarito and Melinda A. Solmon (US)	2006	“This study uses poststructuralism as a lens to investigate how studentsʼ construction of meanings around the body varied by gender and race, and how bodily meanings related to studentsʼ participation in physical education classes” (Azzarito and Solmon, 2006).	Quantitative	HQ
Making Chinese-Canadian masculinities in Vancouver’s physical education curriculum	Brad Millington, Patricia Vertinsky, Ellexis Boyle, and Brian Wilson (Canada)	2008	“Our paper illustrates how males of Chinese descent in British Columbia (BC) have historically been victims of overt and subtle forms of discrimination, and describes how racism is and was integrally linked to notions of class, gender and the body. Highlighted in the historical overview are issues around race and masculinity for Chinese males as they existed (and still exist) in the BC educational system, especially in sport-related and physical education (PE) contexts” (Millington et al., 2008).	Theoretical work	
Aboriginal youth and their experiences in physical education: this is what you have taught me	Joannie M. Halas (Canada)	2011	“In this paper, I incorporate Shawn Wilsons’ (2008) understanding of relationality and relational accountability as I tell the story of my own involvement with Aboriginal youth and the lessons they have taught me about their experiences in physical education” (Halas, 2011).	Qualitative	HQ
Youths with migration backgrounds and their experiences of physical education: an examination of three cases	Dean Barker, Natalie Barker-Ruchtia, Markus Gerber, Erin Gerlach, Simone Sattler, and Uwe Pühse (Switzerland)	2011	“The broad objective of this paper is to provide insights into how physical education intersects with biographies shaped by migration.” (Barker et al., 2014).	Qualitative	HQ
“Because I am Muslim, I cannot wear a swimsuit”: Muslim girls negotiate participation opportunities for physical activity	Manal Hamzeh and Kimberly L. Oliver (US)	2012	“…we discuss in this paper how 4 Muslim girls (ages 14–17 years) negotiated their participation in opportunities for physical activity” (Hamzeh and Oliver, 2012).	Qualitative	HQ
African immigrant students’ experiences in American physical education classes	Seidu Sofo, John Nandzo, Eugene F. Asola, and Kaduabu S. Ajongbah (US)	2013	“Drawing on a neo-racism theoretical framework, the study examined 17 African immigrant students’ experiences in physical education classes in the United States (US)” (Sofo et al., 2013).	Qualitative	HQ
Brown bodies, racialization and physical education	Katie Fitzpatrick (New Zeeland)	2013	“This article explores how school physical education (PE) can both reinforce stereotyped notions of the brown body as inherently physical while also allowing young people to gain educational success. Drawing on a critical ethnographic study of Māori and Pasifika (Pacific Island) youth in PE in New Zealand, the article explores how the academic status of PE, and its alignment with sport, positions the brown bodies of these youth in problematic and stereotypical ways” (Fitzpatrick, 2013).	Qualitative	HQ
‘Racialized’ pedagogic practices influencing young Muslims’ physical culture	Symeon Dagkas and Lisa Hunter (UK)	2015	“This paper draws on Bourdieu’s social theory in an effort to explore the ways in which the intersectionality of various fields (family, religion and school) and their dimensions (culture and social class) influence young Muslims’ physical culture. Purpose: More specifically the paper examines the racialized pedagogic practices in various fields that influence young Muslims’ dispositions to physical culture” (Dagkas and Hunter, 2015).	Qualitative	HQ
Racial representation in physical education textbooks for secondary schools: Image content and perceptions held by students	María Inés Táboas-Pais and Ana Rey-Cao (Spain)	2015	“The purpose of this article is to examine the representation of race through images that are published in Spanish physical education textbooks for secondary schools and to offer an insight into students’ beliefs related to racial stereotypes in physical education” (Táboas-Pais and Rey-Cao, 2015).	Quantitative	HQ
Understanding young Chinese Australian’s (dis)engagement in Health and Physical Education and school sport	Bonnie Pang and Doune Macdonald (Australia)	2015	“Purpose: while there is a high Chinese student population in Australian schools, little research has been undertaken to understand their needs, experiences and perceptions in schools HPE and sport” (Pang and Macdonald, 2015).	Qualitative	MQ
A figurational analysis of how indigenous students encounter racialization in physical education and school sport	Jacqueline A. Williams (Australia)	2016	“This paper problematizes the concept of ‘biological race’ as one such assumption at three government high schools in Canberra, Australia’s national capital” (Williams, 2016).	Qualitative	HQ
Students’ physical education experiences in a multi-ethnic class	Ingfrid Mattingsdal Thorjussen and Mari Kristin Sisjord (Norway)	2018	“By applying an intersectional lens, our aim is to investigate students’ experiences in a multi-ethnic co-educational PE context. Specifically, we ask how the students’ multiple identities may influence their experiences within PE, and what processes of inclusion and exclusion are revealed through their narratives” (Thorjussen and Sisjord, 2018).	Qualitative	HQ
Social inclusion in multi-ethnic physical education classes: Contextualized understandings of how social relations influence female students’ experiences of inclusion and exclusion	Ingfrid Mattingsdal Thorjussen (Norway)	2020	“This paper aims to examine how female students’ diverse backgrounds influence their positioning among classmates and to investigate how inclusion and exclusion in PE can be understood in light of social relations in multi-ethnic classes” (Thorjussen, 2020).	Qualitative	HQ
Inclusion and exclusion in multi-ethnic physical education: An intersectional perspective	Ingfrid Mattingsdal Thorjussen and Mari Kristin Sisjord (Norway)	2020	“In this article, based on students’ stories from a multi-ethnic PE context, an intersectional perspective is used to investigate how processes of inclusion and exclusion are revealed” (Thorjussen and Sisjord, 2020).	Qualitative	HQ

## 3 Results

A total of 5,213 publications were identified, of which 16 were eligible for inclusion. Two used a critical analysis approach, 12 used qualitative methods, and two used a quantitative approach. All publications were published in English, no German publication was found. Different approaches to defining racism were proposed. Every publication was assigned to the top two of the three quality categories using CASP and MMAT. During thematic analysis, recurring patterns and connections are identified through repeated review and reflection on the data. These patterns are then categorized as themes. Through this process five themes could be constructed: (1) “How racism is understood,” describing and interpreting the underlying concepts of racism in the included publications. (2) “Racism in PE: What students experience.” Students reported their experiences of racist discrimination in PE, sports, and everyday life. Researchers often classified them as racial stereotypes, prejudices, and everyday racism; (3) “What PE teachers can and should do?” The articles contained ideas and demands regarding how teachers could meet the challenges of racism in PE. These ranged from concrete recommendations for teachers to demands for nationwide teacher training and curriculum reform; (4) “What institutions can and should do?” The articles offered suggestions and demands on how organizations may tackle the problem of racism in PE; and (5) “What researchers can and should do?” The reviewed papers provided recommendations according to how researchers, particularly sports scientists, should approach the issue of racism in PE in future research.

### 3.1 General overview

Table 2 shows the results of the systematic review.

## 4 Main research findings

This section focuses on the results and discussion of individual themes, particularly emphasizing students’ experiences as it contained most codes in the analysis. Please note that some quotes in the next section are racist. Though they are problematic, the goal is to critically examine them. This is a fundamental issue in anti-racist work to fight against racism: racism needs to be identified and made visible, so that it can be taken apart and understood. Further “simultaneous learning and unlearning of ideological underpinnings that organize the dominant social relations” is needed (McDermott, 2014, p. 211). As part of the thematic analysis, the presentation of the main research findings is combined with the discussion. To distinguish between the interpretations and conclusions of the publications included and our own, we use phrases such as “we” or “our” when applicable.

### 4.1 How racism is understood

As introduced, studies that refer to racism or racialization were included in the systematic research. Thus, it should be noted that three studies define racism explicitly and name an underlying understanding (Hamzeh and Oliver, 2012; Sofo et al., 2013; Williams, 2016). For example: “Neo-racism served as the theoretical framework for the current study” (Sofo et al., 2013, p. 61). In the following discourse, the authors expound upon the diverse dimensions of neo-racism. Eight studies refer to the racialized ‘other’ (Azzarito and Solmon, 2006; Millington et al., 2008; Hamzeh and Oliver, 2012; Dagkas and Hunter, 2015; Pang and Macdonald, 2015; Táboas-Pais and Rey-Cao, 2015; Thorjussen and Sisjord, 2018; Thorjussen, 2020). The description of racist stereotypes (Azzarito and Solmon, 2006; Millington et al., 2008; Fitzpatrick, 2013; Barker et al., 2014; Táboas-Pais and Rey-Cao, 2015; Thorjussen and Sisjord, 2018) and the deconstruction of apparent biological racism can be found in several publications (Azzarito and Solomon, 2005; Fitzpatrick, 2013; Táboas-Pais and Rey-Cao, 2015; Williams, 2016). There is a multitude of other concepts to which various publications refer encompassing poststructural analysis (Azzarito and Solmon, 2006), CRT (Barker et al., 2014), manifestations of social racism (Dagkas and Hunter, 2015), institutional and structural racism (*ibid*.), cultural construction (Táboas-Pais and Rey-Cao, 2015; Williams, 2016), intersectional perspectives (Táboas-Pais and Rey-Cao, 2015; Thorjussen and Sisjord, 2018, 2020), individually formulated definitions (Thorjussen, 2020), historical paradigms (Halas, 2011), and postcolonial perspective (Azzarito and Solmon, 2006).

The studies differ significantly in their approaches as well as in the question of whether an understanding of racism, on which the surveys are based, is fundamentally explained. The first recognition could be explained by the fact that racism is a global phenomenon that differs locally (Babacan et al., 2020). Our interpretation is that the variations in approaches among countries may also be traced back to historical and geopolitical influences. For instance, individual immigration figures contribute to diverse, nation-specific migration patterns. Consequently, this phenomenon has the potential to affect the manifestation of racism in physical education settings and how it is perceived. Despite the different concepts and target groups, the authors report on similar experiences of the students, such as the confrontation with racist stereotypes (see section “What students experience”). Nevertheless, it seems important to clarify the understanding of racism precisely because of the multitude of approaches and definitions. However, this is only found in three studies and should be taken into account in future research.

### 4.2 What students experience

Many articles included in this work feature students’ experiences of racism in physical education. The majority of the articles focus on students affected by racism. The observation that there seems to be a hidden curriculum is present. Intersectional connections between forms of discrimination were also considered. In many of the included publications, the theme presented now plays a dominant role.

Students affected by racism shared negative racist prejudices that they were confronted with in PE classes and in school. In Millington et al.’s (2008) publication, a student is quoted referring to his classmates as ‘the little Asians who study all the time’ (p. 195). The authors of the publication interpret this as invoking the damaging historical stereotype that Asian boys are unsuited for physical education. These negative stereotypes often led to a lack of participation in PE which is for example described by Sofo et al. (2013, p. 66): “The [African] immigrant students perceived their PE classes to be profoundly negative, due, in part, to their perception of negative stereotyping by their peers and their teachers’ lack of culturally responsive pedagogical skills.” Another example of racist stereotypes can be found in Williams (2016, p. 12), in which a teacher describes Aboriginal students using the following words: “As a culture I believe they are pretty active … traditional people that they. … are out there walking or going ‘walkabouts’ or they are … chasing … food or whatever (teacher 3B).” Based on the prejudices stated in this study, our interpretation is that the teacher had a further positive but nevertheless racist expectation due to the culture of Aboriginal students in PE. This was also perceived by the affected students in the same study: “Because they (HPE teachers) just … expect … all the Aboriginal kids … ‘he only plays footy or League’ (student 2C)” (*ibid*., p. 13). We could discern that the stereotypes encountered by students varied based on their respective ethnic groups; however, nearly all the included publications corroborated their prevalence. For instance, McGuire and Collins (1998), Millington et al. (2008), Pang and Macdonald (2015), and Williams (2016) reported the stereotypes of Asian-read people; Halas (2011), Fitzpatrick, (2013), Barker et al. (2014), and Dagkas and Hunter (2015) outlined the detrimental experiences of indigenous students; Azzarito and Solomon (2005) and Sofo et al. (2013) expounded upon the stereotypes encountered by Black students; Hamzeh and Oliver (2012) provided an account of the stereotypes encountered by Muslim-read students; Thorjussen and Sisjord (2018), Thorjussen (2020), and Thorjussen and Sisjord (2020) offered a more comprehensive overview of the stereotypes and prejudices observed in multi-ethnic classroom settings. These findings suggest from our perspective that individuals who experience racism are subject to discriminatory stereotypes and prejudices from their peers and educators in the context of PE. Further, Bottiani et al. (2020) showed that racial discrimination was significantly associated with lower school involvement and increased disconnection in a comprehensive qualitative study. In our opinion it is reasonable to assume that similar effects also apply to PE. Other examples of racism included microaggressions, name-calling, and exclusion. As a student reported: “…You hear stuff, sometimes in playing sport, they’ll say you are Asian and you cannot play sport, they like to start things like that” (Pang and Macdonald, 2015, p. 10). Another student shared his experience of racist isolation: “On my first day in school here in the United States, I [Black] felt isolated by the American kids. In the classroom, none of the children wanted to sit by me or even near me. Some of them made comments like ‘it stinks over here’” (Sofo et al., 2013, p. 63). In this way we see, that students were exposed not only to stereotypes, but also to deep racial hostility.

Students’ experiences were highly inter-individual, even among different racialized groups: “Through the three students’ narratives, the study has demonstrated the complexity of how students’ multiple identities intersect and shape their PE experience” (Thorjussen and Sisjord, 2018, p. 703). Similar inter-individual findings were also reported by Millington et al. (2008), Hamzeh and Oliver (2012), Dagkas and Hunter (2015), and Pang and Macdonald (2015). We assume these individual differences encompassed not only the perception and evaluation of the forms of discrimination presented, but also how they were dealt with. This leads to the conclusion that students’ requirements may be similarly individualized. Programs aimed at supporting and combating racism in affected individuals would also need to adopt a partially individualized approach.

Some of the included studies also describe a hidden curriculum that students must navigate. For example, citing Yosso’s (2002) work, Pang and Macdonald (2015) described a curriculum in which dominant white privileges were hidden. Similar findings regarding the existence of a hidden curriculum and hidden character of racism could be found in other studies (Macdonald et al., 2009; Douglas and Halas, 2013) that specifically addressed white supremacy or referred to it as a whitewashed curriculum (Flintoff et al., 2015). This perspective could also be observed in teachers’ views, as noted by Halas (2011). From our point of view, a hidden curriculum refers to the unspoken or implicit lessons, values, and behaviors that students learn in school through the social environment, interactions with teachers and peers, and school culture. Referring to Flintoff et al. (2015), these hidden norms are “whitewashed.” An example to illustrate these assumptions are “not necessarily vocalized conceptions of ‘good’ or ‘bad’ schools for teaching practice, reflected our stereotypical (racist) assumptions that children from ethnically diverse schools would be ‘less able’ in PE, or ‘more difficult’ to teach” (Flintoff et al., 2015, p. 562). For example, Thorjussen and Sisjord’s (2018) report indicated that racial issues continued to be “silenced” and that the [white] majority culture was reflected in PE activities, with racial issues sidelined. We interpret “silenced” in that matter as the discourse being marginalized, suspended or ignored. For example, in terms of action. Additionally, Barker et al. (2014) described PE as a discursive practice that produces an “other” and criticizes manifestations of cultural inequality within PE. This refers from our perspective to the understanding that certain people or groups are different outside the norm, which can lead to them being marginalized or stigmatized, reinforcing stereotypes. In addition to racial incidents in PE, structural racial discrimination also manifested itself.

Intersectional forms of discrimination played an important role in many of the included studies. Azzarito and Solmon (2006, p. 78) conclude: “For White girls, the ‘perfect body’ was gendered, but for [Black girls] the ‘perfect body’ was gendered and racialized; their viewpoints are representative of double discrimination.” The following studies (Azzarito and Solomon, 2005; Azzarito and Solmon, 2006; Millington et al., 2008; Halas, 2011; Hamzeh and Oliver, 2012; Pang and Macdonald, 2015; Williams, 2016; Thorjussen and Sisjord, 2018; Thorjussen, 2020) examined race and gender, (Hamzeh and Oliver, 2012; Dagkas and Hunter, 2015) race and religion, and (Dagkas and Hunter, 2015) race-related economic capital. However, the focus was primarily on the intersection of race and gender, as “the findings reveal that gender was the most significant factor in the girls’ stories of inclusion and exclusion in PE” (Thorjussen, 2020, p. 1). The author concluded that gender overshadows other differences in PE. To comprehensively understand individuals’ experiences, considering intersectional relationships and avoiding isolating racial discrimination is crucial. In addition to the negative experiences and relationships described, the articles also reported students’ resilience and coping strategies. For instance, the students often reported parental support as an important protective factor (Sofo et al., 2013). Furthermore, interviews with students showed that they resisted the marginalization of minority cultures by making the issue more general (Thorjussen and Sisjord, 2018). In another study, Sofo et al. (2013, p. 65) pointed out that “First, most of them ignored the American students who made fun of them. Rather, they focused on their main objective for being in school — to get a good education.” Success in PE served as a protective factor against discrimination in other subjects (Fitzpatrick, 2013). Despite their importance, these factors were not mentioned in most of the included studies. From our perspective, it is crucial to prioritize these perspectives in the future and work toward strengthening the protective factors in the long term. It would be desirable if these experiences of resilience factors were not just occurring, but if there were safe spaces and contact persons for this purpose within institutions. This would serve to empower individuals affected by racism. Nevertheless, the treatment of the root cause of racism must remain in focus. Further the German large-scale quantitative study, “Racist Realities,” showed that people affected by racism in particular take action against racism in Germany. People aged 14–18 years are particularly active against racism (Seidler, 2022). To support the statement of Thorjussen and Sisjord (2020, p. 62) “to provide students with culturally relevant educational experiences and to develop students’ critical awareness of how culture and ethnicity shape physical culture,” the anti-racist potentials of all students should be used and further strengthened.

According to our understanding, findings clearly illustrate that individuals experiencing racism in PE encounter discriminatory stereotypes and biases from peers and educators. Examples of racial discrimination include microaggressions, name-calling, insults, and exclusion. Structural racial discrimination is also evident through a hidden curriculum. To gain a comprehensive understanding of experiences, intersectional relationships should be considered without focusing solely on racial discrimination. Safe spaces for students affected by racism are needed and the anti-racist potential of all students need increased attention for targeted individual empowerment. Following Dei (2001, p. 139), one approach to support students in their reflection process could be that “all learners can start begin where they are at by striving for knowledge of their own intersecting and interlocking racial, gender, class and sexual identities.”

### 4.3 What PE teachers can and should do

The articles provide recommendations and suggestions on how teachers might address the issue of racism and anti-racism in PE. These range from calls for comprehensive teacher training and curriculum improvements to specific suggestions for teachers, covering awareness, teacher attitude, teaching concepts, reflexive teaching, and teacher training.

In physical education, the racist experiences of Asian heritage students, as concluded by McGuire and Collins (1998), are often not noticed by teachers: “Personal racism-often occurring as racist abuse directed at AH [Asian heritage] students during games and not usually noticed by the teacher” (*ibid*., p. 81). In our eyes, this suggests that PE teachers should be aware of such instances of racism as a basis and prerequisite for possible interventions. In addition to discrimination, the students reported racist incidents caused by their teachers: “During elementary and junior high I went to a racist school and the teachers were racist. My gym teacher was like that” (Halas, 2011, p. 17). However, some teachers wished to empower cultural diversity and seized it as an opportunity: “When we acknowledge and affirm our students’ racialized identities, we also unveil opportunities to incorporate our students’ rich cultural knowledge within our classes” (*ibid*., p. 16); this emphasizes the possibilities of a diverse student body. Understanding diversity as an opportunity could succeed if teachers work with a student-centered pedagogy. This proposition can be found in a study by Pang and Macdonald (2015), who suggest that by promoting students’ high engagement and self-understanding of their resources, teachers could employ student-centered pedagogies. The qualitative studies in this review concluded that teachers should critically question racist stereotypes in PE textbooks. To paraphrase, the authors demand that “teachers’ initial training should enable them to reflect and become aware of the implications that school materials can have in racial differences” (Táboas-Pais and Rey-Cao, 2015). This demand for PE textbooks can certainly be extended to the entirety of the teaching and learning materials in our opinion.

Some studies concluded that teachers are not well-prepared for a diverse student population: “lack of pedagogical skills responsive to diversity in physical education” (Sofo et al., 2013, p. 64). From this finding, many studies call for additional competences for teachers: “educators need to learn more about Islam” (Hamzeh and Oliver, 2012, p. 331). This awareness of values and norms could, according to our view, serve as a basis for understanding and a more respectful approach to working together. Demands for further training were not only made by researchers but also by teachers: “Consequently, some teachers want more training in multicultural education within the curriculum and anti-racism programs in order to meet the challenges arising from increasing student diversity in Australian schools” (Pang and Macdonald, 2015, p. 2). Moreover, further demands regarding teachers in terms of intersectional competences in relation to race and gender were highlighted (Azzarito and Solomon, 2005). The importance of educators adopting critical intersectional pedagogies that go beyond simplistic and binary perceptions of discriminating patterns was suggested (Thorjussen, 2020).

To summarize our angle, teachers must be aware of racist incidents among students and should not act in a conscious or unconsciously racist way themselves; teaching materials for PE lessons should be reflected on critically; and teachers and researchers should demand further anti-racism training and reflect their own role. We would like to add that teaching is a very demanding and complex profession. At the same time, it is not always easy to identify racism in every situation. From our perspective, it is important that teachers prioritize the perceptions and needs of the individuals affected when a potential incident occurs.

### 4.4 What institutions can and should do

The articles provide recommendations and requirements for institutions such as schools, educational authorities responsible for implementing regulations, and publishers of educational materials.

McGuire and Collins (1998, p. 83) noted that in their specific case: “schools are aware of the need for effective multicultural/antiracist policies, and it is relevant to note that all four schools in this study gave full, unconditional support to the research teams.” They advocated that schools create more adapted PE programs that consider students’ ethnic backgrounds. However, other authors noted that such policies are not always implemented: “Evidently, in BC [British Colombia], (as elsewhere) there is a significant distance between official school policies on anti-racism and ‘everyday’ student–student and student-teacher relations” (Millington et al., 2008, p. 209). The findings of Aveling (2007) exposed that school managers lack of comprehensive understanding of racism, yet they were responsible to implement anti-racist policies. From this observation, we are assuming that future research should focus on the implementation of policies and programs. Challenges in this process as well as solutions and successful implementation should be considered. Other demands from institutions found in the publication from Azzarito and Solomon (2005, p. 43) concerned curriculum: “To reconceptualize the predominant multiactivity sports-based curriculum, the field of physical education needs to open and embrace a complicated dialog across cultures, histories, individual identities and ways of being.” We argue future tasks require shaping this dialog in a participatory manner for effective implementation. As another aspect the majority of PE teachers is White in Western countries in contrast to the divers body of students (Sofo et al., 2013). We are coming to the conclusion that institutions should strive for diverse teachers that could meet the perspectives of the diverse student populations in a global world. This must start with the PETE programs but also include teachers in schools, governmental bodies and research in PE as well.

Specific demands on publishing houses were outlined by Táboas-Pais and Rey-Cao (2015, p. 9), based on the results of their quantitative work: “This study demonstrates the need to raise awareness with regard to the content of physical education textbooks and the importance of working to overcome racial stereotypes associated with sport and physical education.” The authors emphasized that to participate in the enhancement of PE textbooks, publishing firms and educational authorities must be well-aware of their content and impact (Táboas-Pais and Rey-Cao, 2015). In our eyes this could succeed if the publishing houses were aware of their responsibilities and deconstructed racist phenomena.

In summary, according to our understanding, institutions should strive for continued anti-racism efforts, utilizing existing positive programs and initiatives, while also ensuring more effective implementation of anti-racist policies. Institutions should strive for greater diversity among PETE students, PE teachers and researchers. There is a need to redesign physical education curricula to incorporate multicultural diversity, and publishers and education authorities should critically reflect on their content and impact through an anti-racist lens.

### 4.5 What researchers can and should do

It is noteworthy that some researchers reflected on their own positions: “My own position as a non-Aboriginal, white researcher contributed to my hesitation to write this story” (Halas, 2011, p. 5). This transparency could also be found in other authors, but it was not common in all included publications. In the 16 studies examined, 6 authors explicitly articulate their positions, while 10 authors do not disclose their stances. Five first authors self-identify as White (Halas, 2011; Barker et al., 2014; Thorjussen and Sisjord, 2018; Thorjussen, 2020; Thorjussen and Sisjord, 2020), and one as Hong Kong Chinese (Pang and Macdonald, 2015). We assume that the remaining authors are predominantly White scholars. The studies in question involve an examination of children affected by racism as well as those unaffected by it. Nevertheless, a critical observation arises regarding why White scholars, not more scholars who identify with the participants, have the access, power, and resources to narrate the experiences of marginalized students, particularly when seeking to dismantle racism in physical education. This critique is also applicable to the present publication. Blackshear (2022) criticizes that “Black women’s narratives, especially in PE, are narrated and controlled by non-Black scholars who lack the embodied experiences of Black women” (Blackshear, 2022, p. 3). Also, Dei (2014) demands for research and policy to be grounded in the communities that are most impacted by the generation of knowledge in research and policy decisions. We emphasize that future scholarly investigations, in particular, may opt for a divergent approach in this regard.

Social dynamics within PE are marked by hierarchical power dynamics, in which students are stratified based on characteristics such as gender, ethnicity, race, social class, and other differentiating factors (Thorjussen, 2020). “However, more studies are needed to explore the diversity of girls’ PE experiences in a multi-ethnic context” (*ibid*., p. 2). The imperative to undertake additional investigations on the intersection of gender and race was a recurring idea in the articles: “Future studies are needed to investigate how expressions of Blackness intersect with discourses of masculinity and femininity and, ultimately, how these negotiations might influence Black boysʼ and Black girlsʼ participation levels in PE classes and school athletics” (Azzarito and Solomon, 2005, p. 91).

Moreover, Thorjussen and Sisjord (2018) highlight that the fundamental nature of PE is not inherently inclusive. As noted in the introduction to this study, while sport can certainly promote inclusivity, it can also perpetuate exclusionary and discriminatory practices. Therefore, the authors recommend further research to explore this complex and multifaceted phenomenon, with particular attention paid to the experiences of students: “…how their experiences can shed light on processes of inclusion and exclusion in the multi-ethnic, mixed-gender PE setting” (*ibid*., p. 696).

For future research, Millington et al. (2008) deemed that it is essential to include the experience, needs, and wishes of the students: “Incorporating student voice is a necessary, if time-consuming endeavor, but it is one that is particularly challenging considering the variability and diversity of contemporary Asian identities,” (*ibid*., p. 208). In our estimation the heterogeneity discussed in the “What students experience” section not only applies to students who identify as Asian but fundamentally to individuals who are affected by racism. Experiences and needs are constantly individualized in nature. It would be worthwhile to listen to these students and include their perspectives in future research.

The following recommendations are made from our perspective regarding future research: First, authors should critically reflect on and elucidate their perspectives and the perspectives of the participants should ideally also be reflected in the authorship. Second, given that PE is not inherently inclusive, additional studies should scrutinize power dynamics. Third, the intersectional discourse in PE warrants further examination. Fourth, there is a need for a deeper exploration of students’ experiences and needs, owing to the considerable variation among racialized students’ experiences. Fifth, we also need White researchers who have critically reflected on their own position to actively work against racism through research.

### 4.6 Limitations

We identified the following limitations for this article: In terms of the search criteria of the systematic literature search, the keyword combinations and selected databases should be highlighted as other keywords or databases might generate different results. Also, our search only included German and English publications. The included studies had to specifically describe the phenomenon of racism. While other studies dealt with racial discrimination, they did not refer to it as such, especially in the German context. To maintain methodological rigor, we excluded these studies. Since the feasibility of evaluating these studies was beyond the scope of this research and was not within the expertise of the authors, they were not pursued. Thus, it may be possible that more sources that included students’ perspectives were overlooked, which is promising from a research perspective. The themes used in the template analysis were constructed by the first author. As aforementioned, he examined the themes from a White male perspective, who has never experienced racial oppression. To circumvent the potential for unconscious biases and personal preconceptions, the author consistently engaged in self-reflection to identify and address potential blind spots. Additionally, the second author, who identifies as a Black woman, consistently challenged and revised the themes.

We mainly identified qualitative studies conducted in English-speaking countries through this systematic literature review. In general, it appears that there are few large-scale quantitative surveys devoted to racism-critical PE from students’ perspectives, whereas there are promising quantitative and theoretical approaches. Despite Germany’s historical responsibility to act racism-critically (Messerschmidt, 2017), publications on this topic are scarce. Although there is an established and empirically supported German discourse on intercultural education in sports (Möhwald, 2019), it often includes only the racism dimension of culture. Other dimensions of racism, such as religion and phenotypic traits (Cole, 2015), have not been sufficiently considered. According to Nieke (2008), in the recognized theory of intercultural education in Germany, calls for focus point 5 out of 10 to address racism. However, this has not been empirically verified in Germany, as this paper shows. Anti-racism is an important component of intercultural pedagogy. In Europe, there appears to be a proclivity to avoid certain terms like “racism” due to its negative connotations stemming from the Second World War; this practice has been adopted by several European nations (Barot and Bird, 2001). Currently, while some publications deal with anti-racist teaching, none have specifically focused on PE (Fereidooni and Simon, 2020). Nevertheless, as the global phenomenon of racism differs locally (Babacan et al., 2020), there is a clear need for a qualitative and quantitative investigation of students’ perspectives in Germany. This principle applies universally, including those countries where such surveys have not yet been conducted.

As mentioned in detail earlier in “What researchers can and should do?” most of the publications were conducted by White researchers. The demand by Dei (2014), for research to be anchored in the communities most directly affected by the generation of knowledge in both research endeavors and policy formulations, remains unaddressed. We realized that the publications are often missing non-white perspectives from the authorship as they lack embodied experiences.

We suggest that whenever researchers conduct research on racism, their own stereotypes and biases should be reflected, and their positions should be made transparent. In this way, the authors enlighten the readers about their personal positions and possible biases to enable others to make a fair assessment of the credibility and reliability of the research results.

## 5 Conclusion

This study’s findings showed that students affected by racism face racist stereotypes and incidents in PE from their peers and teachers. Stereotypes impact racialized individuals in schools, leading to lower school involvement and increased disconnection (Bottiani et al., 2020). These effects may extend to physical education. Structural racial discrimination, which is evident in the hidden curriculum, could be found in some publications. The findings emphasize the importance of considering intersectional perspectives on discrimination patterns. This demand is discernible in supplementary publications, particularly concerning future investigations (Flintoff et al., 2008; Azzarito, 2016; Dagkas, 2016). In the findings section of this publication (Arneback, 2022) and other publications, it became evident that teachers and institutions were urged to implement anti-racist policies and critically reflect on their content and impact. Moreover, even though students faced discriminatory experiences in almost all the included studies, it was not possible to generate the theme “What students could do.” The research field on PE is shaped dominantly by White researchers (Blackshear, 2022). Further authorship in research should aim to include diverse perspectives that can relate to the embodied experiences of the participants (Dei, 2001). Thus, we are convinced that, future research should focus not only on students’ experiences but also on their needs. Anti-racist programs and policies should not only be considered at a structural level and targeted toward teachers but should also include students.

From our perspective it seems indispensable to conduct a qualitative and quantitative survey, which is missing in Germany so far, on students’ experiences, needs, anti-racist potential, and protective factors in PE, while also considering teachers’ perspectives. This could be a useful basis for future training modules regarding teachers and students, with the aim of deconstructing racism in PE and sports and countering it more effectively.

## Data availability statement

The original contributions presented in the study are included in the article/supplementary material, further inquiries can be directed to the corresponding author.

## Author contributions

LA: Writing – original draft. AG: Writing – review & editing. LZ: Writing – review & editing. TV: Writing – review & editing.
